# Single-cell mass-density measurements using microchannel gradient centrifugation

**DOI:** 10.1038/s41598-026-38872-2

**Published:** 2026-02-13

**Authors:** Richard Soller, Per Augustsson, Rune Barnkob

**Affiliations:** 1 Lund University, Department of Biomedical Engineering, Lund, 22363 Sweden; 2 Microfluidics Solutions, Rome, 00154 Italy

**Keywords:** Biological techniques, Physics

## Abstract

We present a microchannel-based adaptation of mass density-gradient centrifugation for the mass density measurement of cells or microparticles. The workflow consists of three basic steps: Microchannel filling, centrifugation, and microscopy. Microchannel filling and subsequent centrifugation of two liquids with different densities allows the instantaneous, precise, and repeatable generation of a one-dimensional mass-density gradient and the simultaneous sedimentation of cells or particles of interest to a point where their mass density is equal to the gradient’s mass density. We introduce two different methods, calibration particles and tracer molecules, for microscopic mass density readout. We demonstrate the measurement principle by measuring the mass density of yeast cells and show that the method allows the mass density measurement of single cells with a position dependent median uncertainty of 3.3 $\mathrm{kg}\,\mathrm{m}^{-3}$ with a throughput of approximately 16000 cells per hour. This measurement precision is in the range of the best single-cell methods currently in use but with a higher throughput. The method is technically straightforward, robust, and affordable, making single-cell mass density measurements widely available.

## Introduction

Cell mass, volume, and mass density are closely linked to the state of a cell^1^ and are affected by processes such as apoptosis^2^, differentiation^3^, disease^4,5^, and malignant transformations, such as cancer^6^. While the mass and volume of a cell can vary greatly, the mass density is tightly regulated such that cell-to-cell variations in mass density are about 100-fold smaller than the variations in mass or volume^4,7^. This enables monitoring of processes that would not be detectable through mass and volume measurements alone^4,8^. Accurate means to determine single-cell densities $\rho _\textrm{c}$ thus enables identification of cells by their type and state or to measure changes in cells prompted by the application of a drug. Although there have been advancements and innovations in the field of single-cell mass density measurement techniques in recent years^9^, the throughput is often low, ranging from a few cells per minute to a few cells per second^10,11^, and no standard measurement method or commercially available instrument has emerged. This lack of tools limits the exploration and exploitation of cell mass density as a potential biomarker.

Suspended microchannel resonators measure single-cell mass by determining the resonance frequency of a hollow U-shaped structure. Upon the entry of a cell, the mass inside the tube changes slightly, resulting in a shift in the resonance frequency^12^. Single-cell mass density determination requires in this method that the cell volume is known, which necessitates an additional chip-integrated electrical resistance sizing module (Coulter counter) or the ability to perform a second passage of each cell after exchanging the suspending fluid^4,13^. Suspended microchannel resonators can accurately determine single-cell mass density but require complex microfabrication and instrumentation and have a throughput of a few hundred cells per hour^4,8,14^.

Optical methods, such as quantitative phase microscopy, digital holography microscopy, or quantitative phase tomography, estimate the single cell dry mass from refractive index measurements based on that most bio-molecules exhibit a linear relationship between refractive index and concentration^10^. These approaches also require the additional measurement of cell volume to derive the single-cell mass density. Objects that are not translucent or that do not follow an a priori established linear relationship between refractive index and concentration cannot be measured by optical approaches.

A cell’s mass density can also be determined indirectly by recording its velocity during sedimentation, which is then used to deduce the particle’s mass density through the Stokes drag law. There have been some improvements by the use of microfluidic measurement of drag forces^15,16^ or systems that lift and sediment cells by optically induced electrokinetics^17,18^. Also, Neumann et al. demonstrated a method for the determination of nano-particle densities with a CPS disc centrifuge^19^. These methods are difficult to implement with high accuracy as the Stokes drag depends on a cell’s size, shape, and distance to nearby walls, all of which have to be independently measured. This makes high-throughput, single-particle determination difficult and time-consuming with this approach.

With this work, we propose a workflow for single-cell mass density determination based on mass density gradient centrifugation in a rectangular cross-section microchannel. Cells sediment through mass density gradient media to a point where the cell’s mass density equals that of the surrounding medium. This is inspired by a since-long-established approach for determining the suspension average mass density of cells in centrifuge tubes. The position of a layer of cells after centrifugation is then commonly translated to mass density using mass density calibration beads. In contrast to its macro-scale predecessor, the microchannel format enables direct microscope interrogation and classification of cells, rapid formation of a smooth and linear mass-density gradient, short centrifugation times, and robust readout of the single-cell mass density for thousands of cells. We believe this method can make single-cell, mass-density determination broadly accessible.

**Figure 1:**
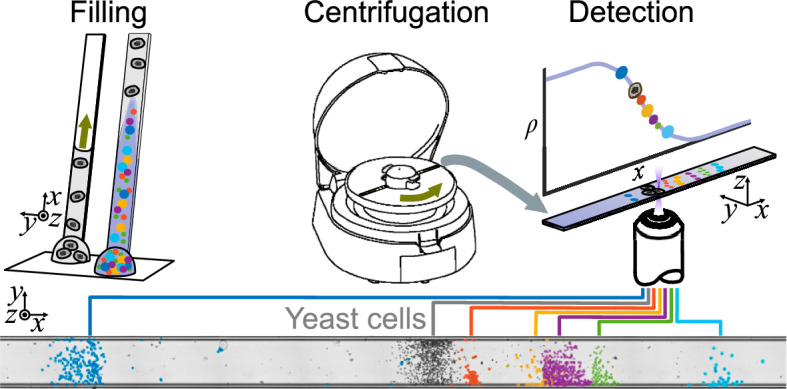
Workflow for single-cell, mass-density measurements using microchannel gradient centrifugation. A microcapillary is filled sequentially with yeast cells in YPD medium and denser 60% iodixanol solution with calibration particles and fluorescent tracer. The capillary is sealed, centrifuged, and scanned with a microscope. The mass-density gradient is calculated by fitting through the calibration particles and the yeast cell densities can be calculated from their positions in the capillary and the local mass-density gradient.

## Results

The workflow, illustrated in Fig. 1, comprises three steps: capillary filling, centrifugation, and detection. Initially, a drop of suspension containing the particles or cells of interest, in a low-mass-density medium, is placed on a piece of parafilm. The opening of a rectangular cross-section capillary is brought in contact with the drop so that the suspension is drawn up by capillary action, filling a part of its internal volume. A second drop, containing a higher mass-density medium (heavy medium) and mass-density calibration beads, is then put in contact with the capillary, and the filling continues until the capillary is full. Under parabolic flow conditions, in combination with molecular diffusion, a continuous mass density gradient forms along the capillary. At first, cells are located in the low mass-density region, while the microparticles reside in the high mass-density region. The capillary is then sealed with wax and centrifuged so cells and particles sediment to their mass-density equilibrium positions. The capillary is scanned with a microscope, and cell densities are computed based on the locations of the cells relative to the reference particles.

### Establishing a mass-density gradient inside the capillary

**Figure 2:**
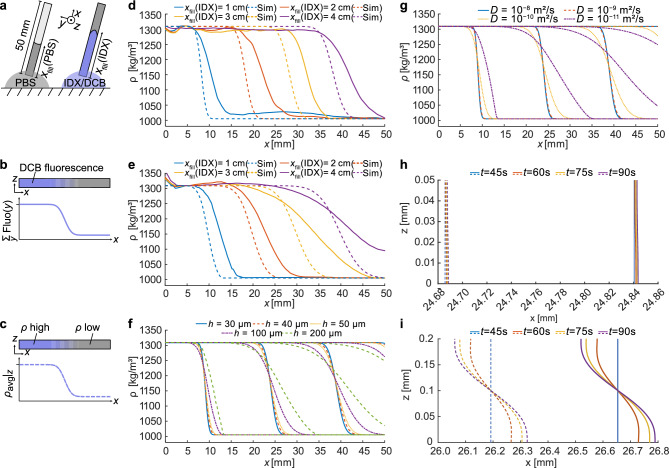
Generation of a mass-density gradient in a microfluidic channel through two-step filling of light (PBS) and heavy medium (Iodixanol IDX with dextran Cascade Blue DCB). (**a**) Schematic representation of the filling process through capillary filling. (**b**) The mass-density gradient is measured experimentally through epi-fluorescence microscopy of the dextran Cascade Blue marker. Experimental images are composed of 43 stitched epi-fluorescence microscopy intensity images. To estimate the mass density gradient $\rho (x)$ along the capillary, marker concentration profiles are extracted from the stitched images by summing fluorescent intensities across the capillary width along the *y*-direction. (**c**) Numerical predictions of the mass density gradient $\rho (x)$ are created from 2D simulations of the iodixanol concentration profiles during the filling process in the capillary. (**d**, **e**) Comparison of experimental (full lines) and simulated (dashed lines) $\rho (x)$-profiles for heavy medium filled to 1 cm, 2 cm, 3 cm, and 4 cm for a capillary height of (**d**) 50 µm and (**e**) 100 µm. (**f**) Numerical $\rho (x)$-profiles for increasing capillary height ranging from 50 to 200 µm. (**g**) Numerical $\rho (x)$-profiles for increasing diffusivity of the solute molecules in the high mass density medium for a 50-µm-high capillary. (**h**, **i**) Time evolution of numerical mass density contours (1100 $\text {kg/m}^3$ and 1110 $\text {kg/m}^3$) for a (**h**) 50-µm and (**i**) 200-µm high capillary after the capillary was filled, centrifuged, and oriented perpendicular to the direction of gravity.

The formation of a longitudinal mass density gradient inside the microcapillary depends on a combination of advection and diffusion at the interface between the light and heavy fluid, Fig. 2 (a). Since the fill time is just a few seconds, while the gradient spans several millimetres, diffusion alone cannot explain the extent of the gradient. Due to the narrow dimensions of the channel, we are in the regime of low Reynolds number fluid physics with dominating viscous forces resulting in laminar flow conditions and parabolic flow profiles with zero flow velocity at the channel walls. The parabolic flow leads to mixing of the liquids due to an enhanced effective diffusion through Taylor-Aris dispersion ^20^.

 Fig. 2(b) shows how intensity profiles are extracted from the stitched epi-fluorescence microscopy intensity images of the experimental concentration field. Since the capillary width is 10 times its height (*h*), we modelled the filling process by assuming a parallel plate geometry in the *x*-*z* cross-section located at the capillary centre. We examined the fluid’s mass density field and extracted mass density profiles corresponding to the experimental profiles,  Fig. 2(c). To examine the evolution of the filling process, experimental and simulated profiles were acquired after positioning the mass density gradient at four nominal positions along the capillary. This was achieved in a sequence of experiments by first filling the capillary with PBS to a pre-determined level and then filling the remainder of the capillary with heavy fluid. For the standard 50-µm-high capillary, Fig. 2(d), the profile has close similarity with an error function resulting in a near-linear gradient spanning approximately 5 mm of the capillary. Despite manual filling results in variations, the generation of gradients is remarkably robust, which is further demonstrated by the similarities of the experimentally obtained gradients with those obtained in simulations. The simulated profiles have a similar shape but are somewhat steeper than the experimental ones. The discrepancy may be associated with the assumption of dilute molecules that is used in the model while in the experiment the molecules are highly concentrated. Increasing the channel height to 100 µm,  Fig. 2(e), leads to a spatial extension of the linear region and simulations show that this effect becomes even more exaggerated when further increasing the channel height, Fig. 2(f). This is a consequence of the Taylor dispersion, i.e. interplay between the deformation of the fluid interface due to the parabolic flow and the radial diffusion. For higher capillaries, the advective dispersion dominates such that the effective diffusion constant becomes $D_{\text {eff}}\propto V_0^2h^2/D$ where $V_0$ is the characteristic flow velocity ^20^. This leads to the perhaps non-intuitive result that the spatial extent of the mass density gradient increases for decreasing diffusivity (*D*) of the solute molecules,  Fig. 2(g).

Since the capillary is oriented horizontally during microscopy, it is important to understand if gravity can lead to a transport of fluid along the capillary due to sedimentation of the heavy fluid underneath the lighter one. Therefore, we performed simulations where the extent of the gradient was monitored over time after the influx of fluid had been stopped and the capillary had undergone centrifugation. In particular, we studied the change of the mass density contours at 1100 $\text {kg}\,\text {m}^{-3}$ and 1110 $\text {kg}\,\text {m}^{-3}$. Fig. 2(h) shows that for a capillary height of 50 µm, the gradient is diffusion dominated and virtually unchanged over 1.5 min, while Fig. 2(i) shows that for a 200 µm capillary, the gradient flattens with time due to a gravity-induced rotational flow in the *x*-*z*-plane. Non-stuck suspended particles and cells can be assumed to follow such flows, and, since the density field is not invariant in *z*, this introduces an uncertainty of $\sim$ 5 $\text {kg}\,\text {m}^{-3}$ when predicting the density of an object based on its *x* position. Therefore, it is crucial to keep the capillary height low and cell experiments were thus conducted on the standard 50-µm-high capillary.

### Cell and particle sedimentation

**Figure 3:**
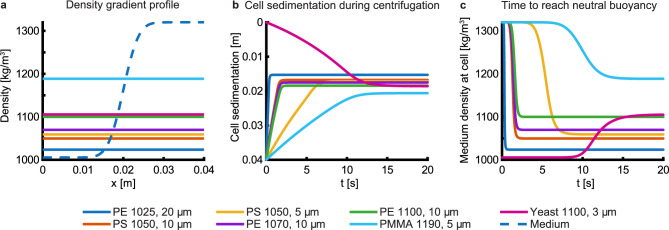
Simulated cell and particle sedimentation. (**a**) Profile of the density medium along the capillary modeled as an error function (dashed). Cell and calibration particle densities are indicated by horizontal solid lines. Materials of particles, measured particle densities, and size ranges are shown in Table 1. (**b**) Simulated particle and cell positions during centrifugation vs time. (**c**) The density of the medium at the location of a particle when approaching neutral buoyancy vs time.

After filling, the capillaries are sealed at both ends with wax to prevent leaking during centrifugation. The capillaries were then centrifuged for 90 s at 12000 rpm in a hematocrit capillary centrifuge, resulting in an average centrifugal acceleration along the capillary of $\sim 5000$ g. Simulated sedimentation of cells in the mass density gradient shows that cells of diameters larger than 3 µm reach their points of neutral buoyancy within 20 s, Fig. 3. The resulting axial force pushes cells and reference particles to their positions of neutral buoyancy. During centrifugation, the rotor temperature increases 1$^\circ \!\text {C}$. This small shift leads to only a slight shift in the mass density of the medium and cell damage due to heating can be ignored.

**Figure 4:**
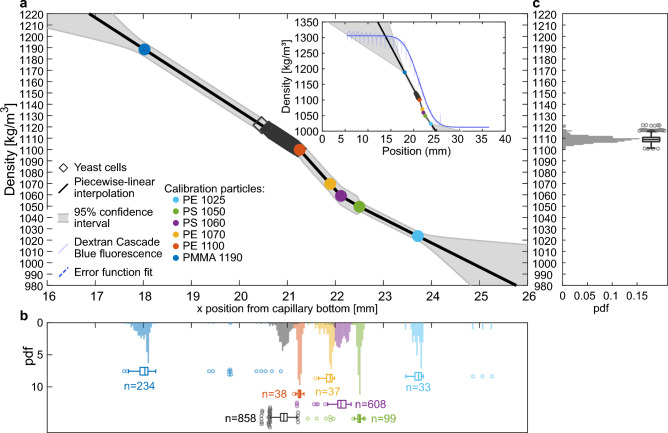
Single-cell, mass-density determination. (**a**) Piecewise-linear interpolation between calibration particle *x*-position and mass density. The insert shows the gradient obtained from the fluorescent tracer molecule dextran Cascade Blue with error function fit. (**b**) Distributions of *x*-positions of calibration particles and yeast cells. (**c**) From interpolation calculated yeast cell mass density distribution.

### Determination of the mass density of single cells

To associate the *x*-position of a detected cell with its mass density, calibration particles of known narrow distributions of densities were added to the mass density modifier suspension before preparing the gradient. After centrifugation, the microcapillary was imaged along its length by standard bright field microscopy. From these images, all cells and particles were identified and their locations in *x* and *y* were recorded by manually clicking on the centre of each calibration particle or cell. Fig. 4 (a) shows the mean locations of the yeast cells and the six different calibration particles for one analyzed capillary and Fig. 4 (b) shows their distributions in *x*. Some cells are localized across the width of the capillary, while some are localized close to the capillary side walls, Fig. 1.

The fluorescence intensity measurement shown in Fig. 4 (a, insert) indicates that, in the investigated range, the mass density of the medium depends linearly on location along *x*. For beads, the reference range $\rho _1$ to $\rho _2$, i.e. the range defined by the sink-float preparation method, is maximally 1.7 $\text {kg}\,\text {m}^{-3}$ (Table 1), whereas the particles *x*-positions are spread wider along the density gradient than the spread of their density fraction would suggest, which we think can be attributed to wall interactions.

The positions of calibration particles were used to establish a piecewise linear density gradient through local gradient estimation between adjacent bead types. The estimated measurement uncertainty of the yeast cells was 3.3 $\text {kg}\,\text {m}^{-3}$ (median of all individual cell measurement uncertainties, interquartile range (IQR): 3.1 - 4.8 $\text {kg}\,\text {m}^{-3}$), with 90% of cells having uncertainty below 6.2 $\text {kg}\,\text {m}^{-3}$. With median measurement uncertainties of 3.3 $\text {kg}\,\text {m}^{-3}$, our method approaches the 1 $\text {kg}\,\text {m}^{-3}$ obtained for yeast with a suspended microchannel resonator^14^. The positions of the calibration particles turned out to be not congruent with the fluorescent intensity gradient obtained from the diffusion tracker dextran Cascade Blue MW 3000 Da, Fig. 4 (a, insert), indicating a systematic over-estimation of cell densities in that approach. The densities of each single yeast cell in a capillary can be calculated with piecewise interpolation from their *x*-position in the capillary, Fig. 4 (c).

**Figure 5:**
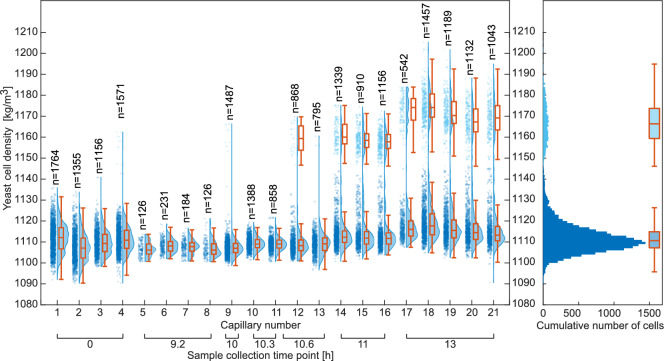
Yeast cell mass density distributions for single capillaries grouped by sample collection times. The histogram on the right cumulated densities for all cells from all measurements. There are two populations: a more numerous, less dense population and a smaller, higher mass density population. The two populations were mathematically separated and boxplots for each are shown on the right.

We then used microchannel gradient centrifugation to measure the mass density of 20677 yeast cells across 21 capillaries (Fig. 5). The median yeast cell mass density is stable for many hours, with a median cell mass density showing a decrease in variance from 4.6 $\text {kg}\,\text {m}^{-3}$ to 2.4 $\text {kg}\,\text {m}^{-3}$ during the first 9 hours. At 11 hours, a new population with a higher mass density appears which we speculate may be dead cells. If the cell wall of a yeast cell ruptures, the mass density medium can diffuse into the cell, leading to increasing buoyant mass density, till it reaches its equilibrium mass density in the mass density gradient. The more numerous lower mass density population has a median mass density of 1111.6 $\text {kg}\,\text {m}^{-3}$(SD: 6.9 $\text {kg}\,\text {m}^{-3}$, IQR: 1106.5 - 1118.5 $\text {kg}\,\text {m}^{-3}$) from a total of 18723 cells across 21 capillaries. This is in agreement with the mass density of yeast measured with other methods: 1102.9 ± 2.6 $\text {kg}\,\text {m}^{-3}$ with a suspended microchannel resonator ^14^, 1040-1130 $\text {kg}\,\text {m}^{-3}$with optically induced electrokinetics^17^, or 1119 ± 11 $\text {kg}\,\text {m}^{-3}$with magnetic levitation^11^. The later-appearing, denser, and smaller population has a median mass density of 1166.8 $\text {kg}\,\text {m}^{-3}$(SD: 9.8 $\text {kg}\,\text {m}^{-3}$, IQR: 1159.3 - 1173.8 $\text {kg}\,\text {m}^{-3}$, 1,954 cells across 12 capillaries) and is closer to previously reported data for the dry mass density of *S. cerevisiae* cell walls of approximately 1180 $\text {kg}\,\text {m}^{-3}$^21^. This observation supports the idea that this population consists of dead hollow cells. Measurement uncertainties vary from capillary and are dependent on the position of the particle or cell along the density gradient. Measurement uncertainty and confidence intervals for each capillary are shown in Supplementary Fig. 1.

Filling, centrifugation, and microscopy of 4 capillaries takes approximately 15 minutes, resulting in a throughput of ca. 16000 cells per hour. This did not include the time for analyzing the images.

## Discussion

We have demonstrated the feasibility of a microchannel-based adaptation of mass density-gradient centrifugation and the application of the method for the mass density measurement of cells. Our results show the ability of our method to measure the mass density of single yeast cells with a median uncertainty of 3.3 $\text {kg}\,\text {m}^{-3}$,which is smaller than the observed biological variation of 6.3 $\text {kg}\,\text {m}^{-3}$(standard deviation, IQR = 7.7 $\text {kg}\,\text {m}^{-3}$) and much smaller that the difference between the two cell populations (peak separation 55.7 $\text {kg}\,\text {m}^{-3}$). This is still a few times higher than the uncertainty of 1 $\text {kg}\,\text {m}^{-3}$achieved with a suspended microchannel resonator but around the same magnitude as other mass density measurements^22^. This is sufficient to detect, for example, the difference in density between different types of cells^11^, the between sickle cell and non-sickle cell red blood cells^23^ or T-cell activation^24^, but may not be precise enough to differentiate density changes due to drug response^24^ or mitotic swelling^25^.

We investigated two approaches to reading out the mass densities and we found the marker-bead approach to be more accurate. While fluorescence intensity mapping yields a very similar profile, the profile is slightly shifted, leading to a systematic error. The main reason we believe is that the diffusivity of the iodixanol and the dextran Cascade Blue is different. This can cause the mass density field to develop at a different rate than the fluorescence intensity field. It is further complicated by the fact that the solution is far from dilute, and thus the diffusivities can be expected to be concentration-dependent. Second, the fluorescence intensity of the fluorescent dextran tend to be higher in water than in iodixanol. Assuming for instance that dextran diffuses faster than iodixanol, their proportions will change, and thus the intensity would increase beyond what can be established by a standard curve, or vice versa. Other density modifiers like Ficoll, used together with fluorescent-labeled molecules of the same type and length as fluorescent concentration markers, may show congruent density and fluorescence gradients. However, since molecular chain length, as well as the degree of labeling, can vary, one cannot assume gradient congruency without using calibration particles.

While we are confident that the pre-sorted mass density marker beads have a mass density range of $\sim$1 $\text {kg}\,\text {m}^{-3}$ (Table 1), there are several possible sources of error. First, as the number calibration particle types of different defined densities is small and the underlying function is not known, the fit to the density gradient is limited. A linear fit using a York^26^ weighted orthogonal distance regression yielded reduced chi-squared values ${\chi _{\nu }}^2 \gg 1$ and residuals that revealed a systematic bias and non-linearity of the density gradient between calibration points. Second, particle populations were spread broader than expected. Across 21 capillaries, particle populations showed a standard deviation of 3 to 17 $\text {kg}\,\text {m}^{-3}$ with (IQRs 2 - 11 $\text {kg}\,\text {m}^{-3}$). Since the mass density gradient appears smooth, we hypothesize that the spread could be caused by wall interactions. During centrifugation, until the particles or cells reach their isopycnic point, they will also be subject to the Euler force, during the acceleration and breaking, and the Coriolis force, throughout, both of which will move the sedimenting object towards the rear wall relative to the direction of rotation. This assumption is supported by particles sometimes aggregating at one *x*-*z* wall of the capillary. This inhibits imaging and counting. In other cases, we see the particles evenly distributed across the *x*-*y* wall of the capillary, which is ideal for imaging and counting. Particle agglomeration will ultimately limit the capacity of the system since objects that are in contact can be pushed out of their isopycnic point by other objects and identification and counting become uncertain. There are several things that can be explored to reduce the agglomeration. To reduce the effect of the Euler force, the acceleration time at the start and end of the centrifugation can be increased. By using a swinging bucket suspension it can be ensured that the centrifugal acceleration is always directed along the capillary. Wider capillaries is expected to lead to less agglomeration due to the Coriolis effect since particles have more room to deflect. The capillaries are put into compartments in the rotor with the thin edge facing the direction of the rotation. These compartments are, however, too big for them. Sometimes, they may flip up while the centrifuge accelerates to speed, which would impact the wall interactions. The inside walls of the glass capillaries are untreated. If the Coriolis and Euler forces push the particles against the walls while they approach their isopycnic point, they may stop at a point where the forces are in equilibrium but before their isopycnic point. Treating the surfaces of walls and/or calibration particles to prevent interactions or the use of surfactant would lead to smaller variances in the *x*-distribution of calibration particles, but manual treatment of microcapillaries is too cumbersome for disposables.

To increase the accuracy and precision of the measurements, the mass density range can be made narrower by exchanging the heavy and light fluid, or the microchannel dimensions can be chosen so as to achieve a less steep density gradient by increased Aris—Taylor dispersion. Less steep gradients would also allow for a higher capacity as cells would spread out more along *x*.

As illustrated in Fig. 2(g) and (i), the capillary must be shallow enough to have a hydraulic resistance that prolongs gravity-induced bulk fluid flow long enough so that the gradient stays stable during microscopic recording. From Fig. 2(i) showing the effect of gravity on the gradient in a 200 µm capillary, it is evident that a calibration particle flowing at the upper quarter of the capillary would be displaced roughly 0.3 mm from a target cell at the bottom quarter of the channel — this would correspond to a measurement error of $\sim$5 $\text {kg}\,\text {m}^{-3}$. In addition to gravity effects in the horizontal capillary during optical assessment, the Euler force acting on the fluid can potentially cause a non-constant density field across the y-axis. This will be most critical during the final stage of the 25 s break-sequence of the centrifuge. At high angular velocities, the density gradient will be stabilized by body forces but in the last few seconds when approaching zero angular velocity the stabilization vanishes while the Euler force is still present. However, the close confinement by the walls at *z* = 0 and *h* will limit this effect through viscous drag.

Our capillary centrifuge has only one speed setting. While we assume that our centrifugation does not influence the short-term viability of the yeast cells, it could influence more sensitive cells. In this case, shear stresses can be reduced by using reduced speed or programmed centrifugation starting with low rpm when the mass density difference is large and ending with high rpm when approaching the isopycnic point.

Compared to other methods, the throughput of the single-cell mass density readout is a few hundred yeast cells per microcapillary, which currently takes around 15 minutes to process. In an automated system to load and scan a capillary, the main time consumption would be the 1-minute centrifugation step.

## Conclusions

With this work, we have outlined and validated a new method to measure the mass density of thousands of single cells in a workflow that has the potential to be carried out in a few minutes. In this relatively simple approach which can be implemented using equipment that is common in many labs, we measured yeast cell densities in good agreement with previous findings and with an accuracy close to that achieved using microchannel resonators. We think that this approach will make single-cell and particle mass density measurements easier and more accessible, paving the way for cell mass density as a biomarker.

## Methods

### Simulated mass density gradient generation

The mass density $\rho (x,z,t)$ during the capillary fill process was modeled in a COMSOL Multiphysics time-dependent simulation combining the Navier-Stokes (NS) equation through the Laminar Flow module and the convection-diffusion equation through the Transport of Diluted Species module. The equations solved were fully coupled by (i) the mass density and dynamic viscosity entering the NS equation are functions of the concentration of iodixanol from the convection-diffusion equation and by (ii) the convective flow velocity entering the convection-diffusion equation is taken from the flow solved in the NS equation.

To model the gradient generation and as we were investigating transport phenomena far away from the liquid-air interface, capillary forces were neglected and the capillary was initially filled with water. At $t=0$ heavy medium entered at $x=0$ at a constant mean velocity $\langle v_x\rangle = 1~\text {mm}\,\text {s}^{-1}$ such that the nominal position of the interface along the capillary could be calculated as $x=\langle v_x\rangle t$. To model the effect of gravity with time when gravity acts perpendicular to the direction of the capillary, the flow was first stopped and centrifugation was performed by applying a constant acceleration $G=10^4g$ along the direction of the capillary. The gradient $\rho (x)$ is extracted by averaging the mass density field $\rho (x,z)$ along *z*.

### Simulated cell and particle sedimentation

The model takes into account the mass density of the particle ($\rho _\textrm{c}$) relative to its surrounding medium ($\rho _\textrm{m}$) and the local viscosity $\eta$ of the medium. The iodixanol concentration in the capillary was modelled to follow an error function spanning from 0 near the centre of the centrifuge and $60 \%$ at the end of the capillary, Fig. 4 (a, inset). The concentration profile was then translated to mass density and viscosity through fitted polynomials based on a priori measured data, following the procedure described in^27^.

We assign each particle a mass density and size *a* and specify the angular velocity $\omega$ of the centrifuge. Under the assumption that diffusion is negligible during centrifugation, the velocity of a particle at different locations *x* along the radial axis of the centrifuge can be expressed

1 $$u(x) = \frac{2}{9}\frac{{\omega ^{2} a^{2} \left( {\rho _{{\mathrm{c}}} - \rho _{{\mathrm{m}}} (x)} \right)}}{{\eta (x)}}x.$$

The radial position vs time of the particle relative to the centrifuge’s centre of rotation was then computed based on Eq. (1) using an explicit Runge-Kutta formula which is implemented in the MATLAB standard routine ode45^28^.

### Preparation of mass density calibration particles

**Figure 6:**
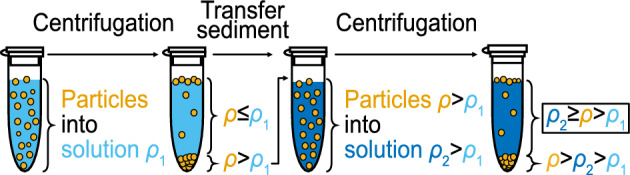
Procedure for preparation of the calibration particles by centrifugation and sink-float method.

In a preliminary step, calibration particles with a defined mass density between $\rho _1$ and $\rho _2$ were prepared through centrifugation with a sink-float method (Fig. 6). First, solutions with defined densities were prepared from Milli-Q water with caesium chloride and 1:$10\,000$ Tween 20, and their densities were checked with a mass density meter (DSA 5000 M, Anton Paar, Austria). Dry microparticles (PS 5 µm and PS 10 µm from microParticles, Germany; all others from Cospheric, USA Fig. 1) were then suspended in a solution with a mass density $\rho _1$ and centrifuged at 3000 g for 15 min. Particles with a mass density lower than $\rho _1$ were floating at the surface, particles with a mass density equal to $\rho _1$ were neutrally buoyant, and particles with a mass density higher than $\rho _1$ sedimented to the bottom. The supernatant containing the liquid with floating and neutrally buoyant particles was removed. The sediment was washed twice by resuspension in milli-Q water with 1:$10\,000$ Tween 20 and centrifugation for 5 min at 3000 g, then the supernatant was removed, and the particles dried until all fluid had evaporated. The dried particles were resuspended in a solution of a mass density $\rho _2 > \rho _1$ and centrifuged again. The sedimented particle fraction will have a mass density of $\rho>\rho _2>\rho _1$. The supernatant containing floating ($\rho <\rho _2$) and neutrally buoyant ($\rho =\rho _2$) particles were recovered and washed twice by resuspension and centrifugation in milli-Q water to obtain a particle fraction with a defined mass density of $\rho _2\ge\rho > \rho _1$.

**Table 1:** Mass density calibration particle fractions of different particle types. $\rho _1$ and $\rho _2$ are the densities of the CsCl solutions used to separate them and denote the upper and lower mass density limits of the particle fraction. Materials are Polyethylene (PE), Polystyrene (PS) and Poly(methyl methacrylate) (PMMA).

Particle type	$\rho _1$ ($\text {kg}\,\text {m}^{-3}$)	$\rho _2$ ($\text {kg}\,\text {m}^{-3}$)	*d* (µm)
PE 1025	1023.196	1024.035	20 to 27
PS 1050	1048.665	1050.406	10
PS 1050	1058.800	1059.378	5
PE 1070	1068.889	1070.418	10 to 20
PE 1100	1098.901	1100.707	10 to 29
PMMA 1190	1188.084	1188.813	5 to 20

### Mass density modifying medium and mass density tracer molecules

The heavy medium consists of the mass density modifier, mass density calibration particles, fluorescent tracer molecules, and 1:10000 Tween 20. OptiPrep (Serumwerk Bernburg, Germany) was used as a mass density modifier because of its favourable density-to-viscosity ratio over the density range we used in our measurements. It contains 60% iodixanol, is isoosmotic, and has a mass density of $1320\pm 0.001$ $\text {kg}\,\text {m}^{-3}$. Dried mass density calibration particles were added to the OptiPrep as a calibration standard. We used 400 µg$\,\text {mL}^{-1}$ fluorescent dextran Cascade Blue 3000 MW (Fisher Scientific, Sweden) to trace the concentration of iodixanol. The pair of iodixanol and dextran Cascade Blue were chosen based on their similar diffusion coefficients^27^, $D_\textrm{DCB} \approx 2.2 \times 10^{-10}$ $\text {m}^{2}\,\text {s}^{-1}$^29^ for dextran Cascade Blue and $D_\textrm{IDX} \approx 2.5 \times 10^{-10}$ $\text {m}^{2}\,\text {s}^{-1}$^30^ for iodixanol. Assuming that the distribution of both molecules in the microchannel is congruent during the measurements, the concentration of the mass density modifier at any point in the channel can be determined by determining the corresponding concentration of the tracer molecule by measuring fluorescent intensity. The densities of the base solutions and the resulting solutions were validated using a mass density meter.

### Cell culture and light cell medium

Commercially available fresh compressed yeast (Jästbolaget AB, Sweden) were cultured in yeast extract peptone dextrose (YPD) medium in 75 $\mathrm {cm^2}$ culture flasks at room temperature. Cells were centrifuged at 200 g for 3 min and resuspended in fresh YPD medium before the measurements. Samples were taken from the culture flasks at different time intervals after resuspension in a fresh medium. For measurements, the cell suspensions were diluted 1:20 (0 min sample), 1:100 or 1:50 (9.2 h samples), or 1:10 (all other samples) with YPD medium and measured without washing and resuspending steps using their cell culture medium as the low-density medium. The YPD medium mass density was determined with a mass density meter at 20$^\circ \!\text {C}$. YPD medium has a higher osmolarity than PBS or Optiprep, and sedimentation of yeast cells into a mix of YPD medium and Optiprep may exert osmotic stress on the yeast cells. However, Bryan et al.^14^ showed no volume change by suspending yeast cells in phosphate buffer.

### Capillary filling and centrifugation

500 µm $\times$ 50 µm $\times$ 50 $\text {mm}$ capillaries (VitroCom, USA) were used for cell mass density measurement experiments. To fit into the ServoSpin hematocrit centrifuge (ServoPrax, Germany) rotor, capillaries were shortened to 40 $\text {mm}$. 500 µm $\times$ 50 µm $\times$ 50 $\text {mm}$ and 1000 µm $\times$ 100 µm$\times$ 50 $\text {mm}$ capillaries (CM Scientific, UK) where used for the gradient generation study. 10 µL drops of heavy medium (with calibration beads and tracer molecules) and low-mass-density medium (cell medium with cells for yeast experiments, otherwise PBS), respectively, were pipetted onto parafilm, forming two hemispherical drops. The capillaries were first filled by low mass density medium to a predetermined level by capillary force by bringing the capillary in contact with that drop. Excess liquid on the capillary outside was gently removed to reduce cross-contamination of the fluids. The capillaries’ remaining volume was then filled up from the drop of the dense fluid, and the excess liquid on the outside was again removed.  Before centrifugation, the capillaries were sealed at both ends using wax plates (ServoPrax, Germany). Subsequently, capillaries were centrifuged with the ServoSpin hematocrit centrifuge for 90 $\text {s}$ at $12\,000$ rpm. After centrifugation, the capillaries were kept upright to keep the mass density gradient stable until microscope imaging. Capillaries used for the gradient generation study were not sealed and were immediately imaged. The centrifuge rotor temperature was measured with an infrared thermometer (Fluke 62 Max+, Fluke, USA) with a measurement uncertainty of ±1 $^\circ \!\text {C}$.

### Image acquisition

Microscope images were acquired with a Nikon ECLIPSE Ti2-E fluorescence microscope with an automated stage and a Teledyne Photometrics Prime 95B sCMOS camera. The capillaries were placed on a standard microscopy slide with glued-on stoppers. A focus map in *x*-direction was created before imaging to keep the same focus position in the capillary while scanning along its *x*-axis. Lumencor CELESTA Light Engine solid-state laser light source (Ex 365 nm) was used to excite the fluorescent tracer molecule through a standard DAPI filter set. A CoolLED $p\mathrm {T-100}$ LED white light source was used for bright field imaging. All images were acquired with a Nikon 10 $\times$ N.A. 0.3 long working distance objective.

### Data analysis

Bright-field and fluorescence image tiles were analyzed in MATLAB (R2023a, MathWorks) using Image Processing and Statistics toolboxes. Single images were stitched together sequentially by excluding the overlap (Supplementary files *full_capillary_example1.png* and *full_capillary_example2.png*). Cross-correlation analysis of inter-single-image *y*-shift for each image was used to determine and correct the rotation of the capillary and to correspondingly adjust the cell and particle positions. Capillary walls were located on bright-field images by smoothing column intensity profiles with a Savitzky–Golay filter and identifying “flat” regions as points with a derivative magnitude below 2 % of the profile range. Regions within a fixed margin from each wall (wallcut, specified in $\mu$m) were excluded from fluorescence calculations to avoid artifacts from the rounded capillary corners.

The local effect of the calibration particles on the fluorescent marker molecule gradient was corrected by *x*-direction step-wise adaptive-thresholding of the capillary to identify fluorescent intensity outliers and replacement of their values with median values calculated from the subsection looked at. The fluorescence intensity was averaged across the capillary width (*y*) to obtain a one-dimensional fluorescence intensity profile *I*(*x*) along the capillary *x*-axis. For the gradient generation experiments, vignetting in the raw gradient data in the *x*-direction was corrected by normalization of each image section with an averaged image section calculated from the flat path at the start or end of the gradient, which was then filtered with a moving average calculated with a robust linear regression.

An error function was fit to *I*(*x*) (nonlinear least squares). We assumed that the high and low intensity plateaus at the ends of the capillaries contained the heavy medium with its maximum dextran Cascade Blue and iodixanol concentrations or the light medium of the cell suspension respectively. The normalized error function fit of the intensity gradient was assigned minimum and maximum mass density values according to this reasoning. The normalized fit was mapped to mass density via linear rescaling to the interval $[\rho _{\textrm{low}},\,\rho _{\textrm{high}}]$.

A linear fit using a York^26^ weighted orthogonal distance regression yielded reduced chi-squared values ${\chi _{\nu }}^2\gg 1$ and residuals that revealed a systematic bias and non-linearity of the density gradient. We therefore determined individual cell densities by monotone piecewise-linear interpolation between calibration particles of known density (Table 1). For each calibration particle type *j* ($j=$1..6, ordered along *x*), we determined the median position $\overline{x}_j$ from all particles of that type and assigned the corresponding nominal density $\rho _j$. Between adjacent calibration nodes, we calculated local density gradients $g_k = (\rho _{k+1} - \rho _k) / (\overline{x}_{k+1} - \overline{x}_k)$ for segments *k* = 1..5. For cells with positions *x* between the nodes $\overline{x}_k \le x < \overline{x}_{k+1}$, the density was determined through interpolation as $\rho (x)=\rho _k + g_k(x - \overline{x}_k)$. Densities of cells beyond the calibration range (${<}$1% of cells) with positions of $x < \overline{x}_1$ or $x > \overline{x}_6$ were determined through extrapolation using $\rho (x)=\rho _1 + g_1(x - \overline{x}_1)$ or $\rho (x)=\rho _6 + g_5(x - \overline{x}_6)$, respectively.

Measurement uncertainties were computed from three contributions and combined through standard error propagation as $\sigma _{\rho ,\textrm{cell}}^2 = g_k^2 \sigma _{x,\textrm{cell}}^2 + max(\sigma _{\mathrm {\rho ,L}}^2,\sigma _{\mathrm {\rho ,R}}^2) + \sigma _{\mathrm {\rho ,extrap}}^2$. The position uncertainty $g_k^2 \sigma ^2_{x,\textrm{cell}}$ derives from the manual selection of cells (15 px, equal to approx. 9 $\mathrm {\mu m}$ ), amplified by the square of the local density gradients slope. The uncertainties of the two calibration nodes bracketing each interpolation segment contribute as the maximum of their variances. Node uncertainties were determined from the width of the calibration particle fractions and from the *x*-spread of calibration particles. For each particle type, the positional dispersion was quantified as $\sigma _{x,\textrm{bead}} = 1.4826\,\textrm{MAD}(x_i)$, where the MAD scaling factor converts to a standard-deviation-equivalent measure. The positional spread was mapped into density space using the local gradient at that node via $\sigma _{\rho ,\textrm{bead}} = |g_{\textrm{local}}|\,\sigma _{x,\textrm{bead}}$. Density intervals of the calibration particles ( $(\rho _2 - \rho _1)/2$) were added into each node’s total uncertainty, yielding $\sigma _{\rho ,\textrm{node}}^2 = \sigma _{\rho ,\textrm{bead}}^2 + (\Delta \rho _{\textrm{bracket}}/2)^2$. For the small fraction of cells lying outside the calibration range, an additional conservative extrapolation penalty was added. The extrapolation uncertainty was defined as $\sigma _{\rho ,\textrm{extrap}}^2 = (K \times d_{\textrm{extrap}})^2$, where $d_{\textrm{extrap}}$ is the extrapolation distance in mm and $K = 1.4826\,\textrm{MAD}(g_k)$ is a robust MAD-based estimate of the variability of all local gradients.

The cumulative distribution of cells from all measurements can be separated by applying a kernel density estimation (bandwidth = 1.0 $\text {kg/m}^3$) to identify the valley (antimode) between the two main peaks in the pooled density distribution. Cells were assigned to Component 1 (lower density) or Component 2 (higher density) based on this threshold. Measurement uncertainty and confidence intervals for each capillary are shown in Supplementary Fig. 1.

## Supplementary Information

Supplementary Information 1.

Supplementary Information 2.

Supplementary Information 3.

## Data Availability

Data sets generated during the current study are available from the corresponding author on reasonable request.
