# Supplementary material for: Single-cell mass-density measurements using microchannel gradient centrifugation
Source: Sci Rep. 2026 Feb 13;16:6501. doi: 10.1038/s41598-026-38872-2 (PMC12909970; doi:10.1038/s41598-026-38872-2)
Supplement: Supplementary file 1 — Supplementary Information 1. [file 41598_2026_38872_MOESM1_ESM.pdf]

Supplementary information for:

Single-cell mass-density measurements using microchannel gradient centrifugation

Richard Soller,[a] Per Augustsson,[a] and Rune Barnkob[a,b]

[a] Department of Biomedical Engineering, Lund University, Ole Römers väg 3, 22363 Lund, Sweden. E-mail: per.augustsson@bme.lth.se

[b] Microfluidic Solutions, 00154 Rome, Italy. E-mail: rune@barnkob.com

Supplementary Figure 1: Measurement uncertainty

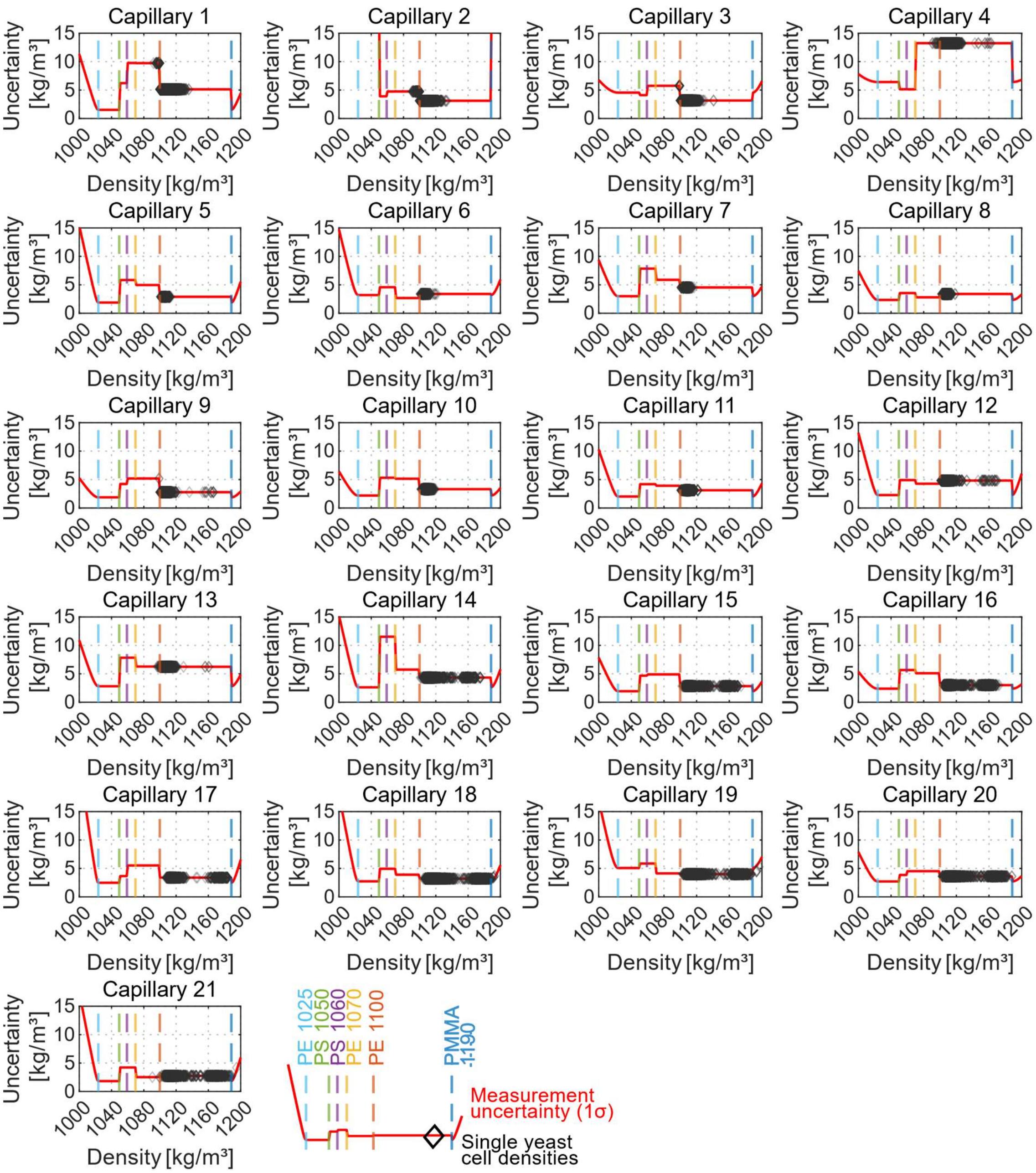

The measurement uncertainty and confidence interval half-width for the capillaries pertaining to Fig. 5 (main article).

Supplementary Figure 2: Gradient before and after Centrifugation

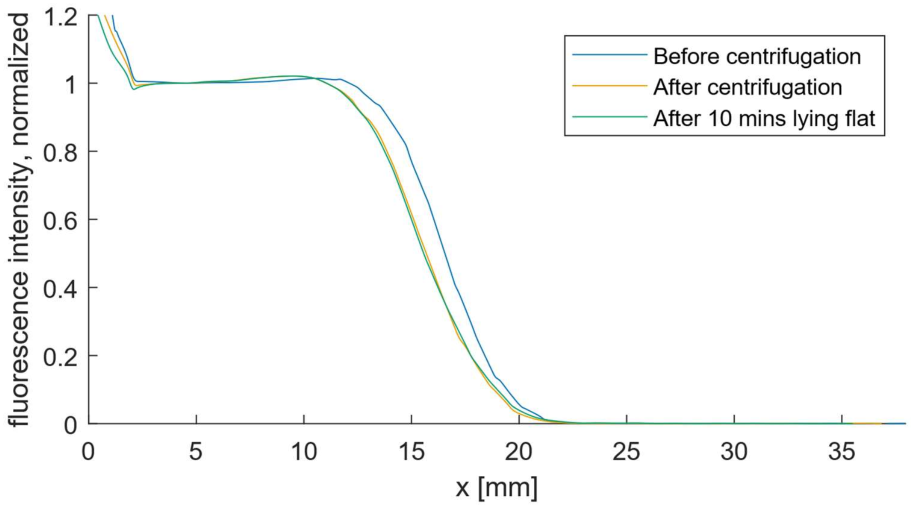

A 500 µm x 50 µm x 40 mm capillary was filled sequentially first to approximately half height with PBS and then to completion with OptiPrep mixed with 400 µg/ml Dextran Cascade Blue 3000 MW (mixed density 1311.2 kg/m^3). The fluorescence gradient in the capillary was microscopically recorded before centrifugation, after centrifugation, and after lying 10 min flat after centrifugation. Image processing as described for other capillaries in the Methods section.
